# Capsaicin Modulates Ruminal Fermentation and Bacterial Communities in Beef Cattle with High-Grain Diet-Induced Subacute Ruminal Acidosis

**DOI:** 10.3390/microorganisms13010084

**Published:** 2025-01-04

**Authors:** Wei You, Haijian Cheng, Xin Hu, Enliang Song, Fugui Jiang

**Affiliations:** 1 Key Laboratory of Livestock and Poultry Multi-Omics of MARA, Institute of Animal Science and Veterinary Medicine, Shandong Academy of Agricultural Sciences, Jinan 250100, China; uv79@sina.com (W.Y.); 98061107@163.com (H.C.); huxin19890803@163.com (X.H.); enliangs@126.com (E.S.); 2 Shandong Provincial Key Laboratory of Livestock and Poultry Breeding, Jinan 250100, China

**Keywords:** capsaicin, subacute ruminal acidosis, beef cattle, ruminal fermentation, bacterial community

## Abstract

This study was developed with the goal of exploring the impact of capsaicin on ruminal fermentation and ruminal bacteria in beef cattle affected by high-grain diet-induced subacute ruminal acidosis (SARA). In total, 18 healthy Simmental crossbred cattle were randomized into three separate groups (*n* = 6/group): (1) control diet (CON; forage-to-concentrate ratio = 80:20); (2) high-grain diet (SARA; forage-to-concentrate ratio = 20:80); and (3) high-grain diet supplemented with capsaicin (CAP; 250 mg/cattle/day). The study was conducted over a 60-day period. The results showed that the SARA model was successfully induced in the SARA group with a high-grain diet. Relative to the SARA group, the addition of capsaicin elevated the ruminal pH from 5.40 to 6.36 (*p* < 0.01), and decreased the total volatile fatty acids (VFAs) from 133.95 to 82.86 mmol/L (*p* < 0.01), aligning closely with the levels observed in the CON group. The addition of capsaicin increased the alpha diversity of ruminal bacteria relative to the SARA group, as evidenced by a lower Simpson index (*p* < 0.05), together with increases in the Ace, Chao, and Shannon indices (*p* < 0.05). Bacteroidota and Firmicutes were the most common phyla across all treatment groups, while *Prevotella* was the predominant genera. The unique bacterial genera (LDA scores > 4) identified within the SARA group comprised *Succinivibrionaceae_UCG-001*, *Succinivibrio*, *NK4A214_group*, *Lachnospiraceae_NK3A20_group*, and *Ruminococcus*, which may serve as potential biomarkers for the diagnosis of SARA. The unique genera associated with the CON group included *Rikenellaceae_RC9_gut_group*, *Prevotellaceae_UCG-003*, and *U29-B03*, while those for the CAP group included *Succiniclasticum* and *Prevotellaceae_UCG-004*. In summary, these results suggest that dietary capsaicin supplementation can limit the adverse effects of SARA through the modulation of bacterial communities within the rumen, thus altering ruminal fermentation in beef cattle.

## 1. Introduction

Rising global meat production demands have fueled the adoption of energy-dense diets by the beef industry in an effort to meet corresponding energy requirements and improve beef cattle production performance [1]. These highly concentrated diets, however, are often enriched for fermentable carbohydrates while containing limited amounts of physically effective fiber [2]. Fermentable carbohydrates, which primarily consist of starch, undergo rapid ruminal fermentation that results in the production of substantial quantities of VFAs. This can rapidly reduce the pH of the rumen, exposing cattle to the risk of developing subacute ruminal acidosis (SARA) [3,4]. SARA has emerged as an increasingly common metabolic disease among cattle, particularly dairy cows with high production levels and beef cattle during the later stages of fattening. This condition can cause a range of adverse effects, such as reduced dry matter intake and digestibility, changes in the composition of the bacterial community found in the rumen, impaired microbial protein synthesis, lower milk yields, decreased milk fat content, changes in the lipid profile of milk, liver abscess formation, higher rates of laminitis, and lower conception rates [5]. SARA is, thus, a pressing threat to the health of these ruminants, incurring substantial economic losses. There has, thus, been growing research interest in establishing nutrition-based preventative strategies that can mitigate the adverse effects of SARA.

The natural alkaloid capsaicin (CAP), also known as trans-8-methyl-N-vanillyl-6-nonenamide (C_18_H_27_NO_3_), is the main active ingredient found in chili peppers (*Capsicum annuum*) [6]. Studies of CAP have revealed that it exhibits diverse biological effects, including antimicrobial [7], antioxidant, anti-inflammatory [8], and gastrointestinal health-promoting effects [9]. CAP has been widely used as a feed additive to improve ruminant health, growth, and production performance. In dairy cows, for example, dietary CAP supplementation has been demonstrated to enhance milk production [10,11], while in beef cattle, it can reportedly increase the average daily gain [12]. In lipopolysaccharide-challenged dairy cows, CAP can limit oxidative stress and augment host immune status [13]. These past studies, however, have largely focused on the post-ruminal effects of CAP, which is often delivered in the form of rumen-protected capsicum oleoresin [14,15,16]. The majority of these studies have also been focused on optimizing dairy cows’ health and milk production [14]. The effects of purified CAP on the ruminal microenvironment, by contrast, remain poorly understood, particularly in beef cattle.

The pathological basis of SARA stems from the loss of homeostatic balance between rumen microbe-mediated fermentation acid production and the absorption, retention, and buffering of these acids [17]. As high-concentrate diets are essential to meet the energy demands of high production performance, the enhancement of salivary secretion may be an effective nutritional approach for limiting SARA risk. This is because the saliva is rich in bicarbonates and phosphates that are capable of neutralizing an estimated 30–40% of the acid generated in the rumen [18]. By specifically activating the transient receptor potential vanilloid subtype 1 (TRPV1), CAP can trigger an increase in paracellular pathway permeability in the salivary epithelium, leading to increased secretory output. Consistently, Cong et al. [19] found that the activation of TRPV1 by CAP can enhance rabbit submandibular gland permeability, leading to an increase in saliva secretion. Comparable findings have also been reported in rats and for the SMG-C6 submandibular gland cell line [20,21]. Based on these prior reports, CAP may confer protection against SARA in ruminants through the enhancement of salivary secretion, thereby preserving ruminal microbial community homeostasis and rumen health.

Therefore, one aim of this study was to determine the changes in the rumen bacterial community using 16S rRNA gene Illumina MiSeq, alongside an assessment of fermentation parameters, including pH, acetate, propionate, and butyrate, in cattle experiencing SARA induced by a high-grain diet. Another aim was to determine the effect of supplementation of CAP on rumen bacteria shifts and the fermentation products in beef cattle with high-grain diet-induced SARA. We postulated that a high-grain diet would influence the composition of the rumen bacterial community and the resulting fermentation products. Additionally, we hypothesized that the addition of CAP could mitigate the negative impacts of SARA by modulating the rumen bacterial community, thereby altering ruminal fermentation processes in beef cattle. The findings from this investigation may offer valuable insights into the potential of CAP for preventing SARA.

## 2. Materials and Methods

### 2.1. Animals, Diets, and Study Design

This study was conducted at the Shandong Lurun Co., Ltd., Dezhou, China (37°13′ N, 116°21′ E, 18.5 m average above sea level). The regional climate is temperate, with a mean annual temperature of 12.7 °C and a mean annual rainfall of 570.2 mm. This study was based on a fully randomized experimental design. In total, 18 healthy Simmental crossbred cattle with good body condition scores (6.3 ± 0.4, where 1 = emaciated and 9 = obese), similar ages (14 ± 1 months) and weights (468 ± 43 kg) were randomized into three groups (n = 6/group). The treatments for these groups included: (1) a control diet (CON; forage-to-concentrate ratio = 80:20; dry matter [DM] basis); (2) a high-grain diet (SARA; forage-to-concentrate ratio = 20:80; DM basis); and (3) a high-grain diet supplemented with 250 mg CAP per cattle per day (CAP). Dietary ingredients and chemical composition of diets are provided in Table 1. The CAP used for this study was purchased from Hubei Hongxin Ruiyu Fine Chemical Co. Ltd., Wuhan, China (Capsaicinoid content ≥ 95%). The entire study was 60 days in length, including a 15-day pre-feeding period and a 45-day feeding period. All cattle were individually housed in stalls with free access to water. Feeding took place twice per day (at 08:00 and 18:00) and feed was adjusted daily to obtain approximately 10% orts (on an as-fed basis).

### 2.2. Sample Collection

At the end of the finishing period, the animals were slaughtered at a commercial slaughter facility, according to standard practices. Ruminal fluid was immediately collected after slaughter on the final day of the study, 2 h after the morning feeding [22]. An equal mixture of ruminal liquid (200 mL) was collected from the dorsal, anterior ventral, medium ventral, posterior dorsal, and posterior ventral sites within the rumen, and squeezed through 4 layers of cheesecloth (pore size: 1 mm). Immediately following collection, the pH of the rumen was measured, and samples were separated into two parts. One portion was stored at −80 °C for future analyses of the ruminal bacteria, while the remainder was used for analyses of volatile fatty acids (VFAs).

### 2.3. Analyses of Rumen Fermentation Characteristics

A pH meter (HI-9126; Hanna Instruments, Woonsocket, RI, USA) was used to assess the ruminal pH. The levels of organic acids (acetate, propionate, butyrate, isobutyrate, valerate, isovalerate) in collected ruminal fluid samples were analyzed via HPLC with a Shodex RSpak KC-811S-DVB gel C column (Shimadzu, Kyoto, Japan), using 3 mmol/L HClO_4_ as a mobile phase, with a 1.0 mL/min flow rate [23].

### 2.4. MiSeq Sequencing

DNA extraction, PCR amplification, and MiSeq sequencing of ruminal fluid samples were performed by Majorbio Company (Shanghai, China). DNA was extracted with an E.Z.N.A.^®^ soil DNA Kit (Omega Bio-tek, Norcross, GA, USA) based on provided instructions, after which DNA quantity and purity were assessed using a NanoDrop 2000 UV–vis spectrophotometer (Thermo Scientific, Wilmington, DE, USA), while 1% agarose gel electrophoresis (AGE) was used to assess DNA quality.

The V3-V4 16S rRNA region was amplified using the 338F (5′-ACTCCTACGGGAGGCAGCAG-3′) and 806R (5′-GGACTACHVGGGTWTCTAAT-3′) primers with a GeneAmp 9700 instrument (ABI, Los Angeles, CA, USA). Following 2% AGE separation, the amplified PCR products were purified with an AxyPrep DNA Gel Extraction Kit (Axygen Biosciences, Union City, CA, USA) and quantified with QuantiFluor™-ST (Promega, Madison, WI, USA).

After purification, amplicon pooling was performed in equimolar amounts, followed by the paired-end sequencing (2 × 300) of these samples with the Illumina Miseq PE300 platform (Illumina, San Diego, CA, USA). The Trimmomatic software (v0.39) was then used as described by Bolger et al. [24] to filter the resultant samples based on quality, eliminating bases that were ambiguous or sequences of poor quality (average quality score < 20). When pre-processing was complete, the merging of paired-end reads was performed with FLASH (v1.2.11), using a minimum overlap of 10 bp and a 2% mismatch error rate [25]. QIIME v1.8.0 was used to denoise the sequences, removing chimeric sequences [26]. UPARSE (v11) was used to cluster operational taxonomic units (OTUs) at 97% similarity, standardizing the OTUs based on the sample with the lowest number of sequences [27]. Taxonomic analyses were performed by using the Silva (SSU138) database for 16S rRNA sequence alignment with the RDP classifier algorithm (v2.13) and a 70% confidence threshold [28]. OTUs that were unique or shared among groups were identified through a Venn diagram analysis. The alpha diversity indices (Ace, Chao, Shannon, Simpson, and Coverage) were assessed with MOTHUR (v1.30.2) [29], while differences among groups were calculated through Kruskal–Wallis H tests and Tukey–Kramer post hoc test with FDR multiple test correction. Beta diversity was estimated through a principal coordinate analysis (PCoA) based on weighted unifrac distances in QIIME (v1.9.1) [30,31], comparing differences among groups through an analysis of similarities (ANOSIM) using 999 permutations. The bacterial taxa that were differentially represented among groups at the phylum to genus taxonomy level (biomarkers) were determined using linear discriminant analysis (LDA) of their effect size (LEfSe) [32] and with an all-against-all multiclass analysis, and had a logarithmic LDA threshold of 4.0. The significant difference among groups in bacteria genus level was identified by a Kruskal–Wallis H test and a Tukey–Kramer post hoc test with FDR multiple test correction.

### 2.5. Correlative Associations Between Ruminal Bacteria and VFAs

An RDA approach at the genus level was used to explore associations between bacteria and fermentation parameters using the *R* software (v4.0.3, package vegan), using the fermentation parameters as explanatory variables. Before modeling was performed, variance inflation factor (VIF) values were used to select explanatory variables, removing those variables with a VIF > 10. Correlations between genus-level microbial composition and fermentation parameters were assessed based on Spearman rank correlations, and the resultant correlation matrix was presented in the form of a heatmap.

### 2.6. Statistical Analysis

Before conducting statistical analyses, the data of ruminal fermentation characteristics were assessed for normal distribution using the Shapiro–Wilk test, and for homogeneity of variance using Levene’s test. Subsequently, the data were analyzed by an ANOVA, utilizing the general linear model (GLM) procedure, in SAS version 9.1 (SAS Institute Inc., Cary, NC, USA). Duncan’s multiple range test was employed to identify significant differences among treatment means. All data are presented as the least squared mean, and effects were considered significant at *p* ≤ 0.05 unless otherwise indicated, with trends being recognized in the 0.05 < *p* ≤ 0.10 range.

## 3. Results

### 3.1. Ruminal pH and Fermentation Parameters

Relative to the CON group, the ruminal pH of cattle in the SARA group was significantly reduced (*p* < 0.001; Table 2), while the concentrations of total VFAs, acetate, propionate, butyrate, and valerate in the rumen were significantly increased (*p* < 0.01). Relative to the SARA group, the CAP group exhibited an increase in ruminal pH together with reductions in the levels of total VFAs, acetate, propionate, butyrate, and valerate in the rumen (*p* < 0.01). Similar ruminal pH and individual VFA concentrations were evident when comparing the CON and CAP groups, with the exception of valerate and isovalerate levels, which remained significantly elevated in the CAP group (*p* < 0.05). The isobutyrate concentrations and the acetate-to-propionate ratio were comparable in all three groups (*p* > 0.10).

### 3.2. 16S rRNA Sequencing Results and Ruminal Alpha Diversity

**Figure 1:**
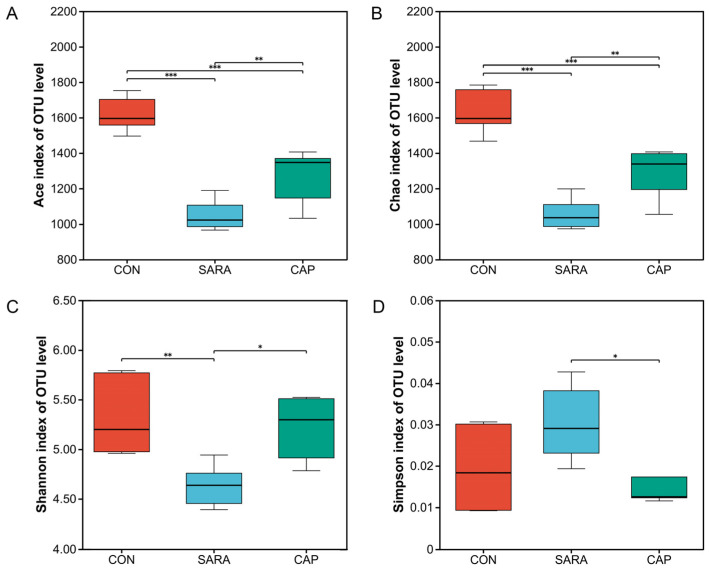
Differences in ruminal bacterial community diversity and richness in specific treatment groups. (**A**) Ace, (**B**) Chao1, (**C**) Shannon, and (**D**) Simpson indices. CON, control diet; SARA, high-concentrate diet; CAP, high-concentrate diet supplemented with capsaicin. * *p* < 0.05; ** *p* < 0.01, *** *p* < 0.001.

The Miseq sequencing of the 18 collected samples yielded 554,112 high-quality sequences (30,784 average reads/sample), which were clustered into 2367 OTUs at a 97% sequence similarity threshold (Supplementary Table S1). Across all treatment groups, Good’s coverage was approximately 0.98, which was consistent with the sufficient sequence coverage. Alpha diversity analyses are presented in Figure 1. The CON group exhibited the highest Ace and Chao1 index values, followed by the CAP group, whereas they were lowest in the SARA group. A lower Shannon index was noted in the SARA group relative to the CON and CAP groups, whereas the opposite was true for the Simpson index (*p* < 0.01).

### 3.3. Composition of the Ruminal Microbiome

**Figure 2:**
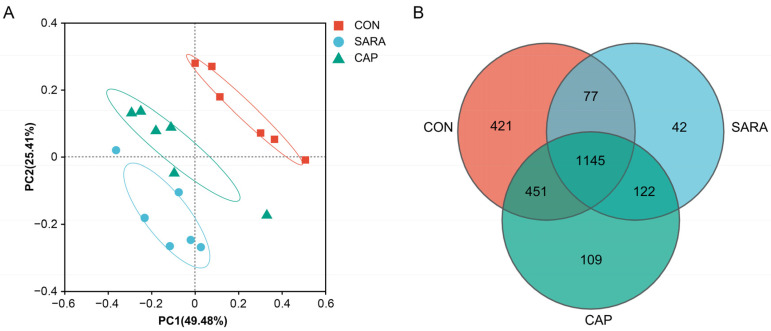
(**A**) Principle coordinate analysis (PCoA) of the ruminal bacteria based on weighted unifrac distances. (**B**) A Venn diagram showing overlapping and unique OTUs in the ruminal fluid samples from the three treatment groups. CON, control diet; SARA, high-concentrate diet; CAP, high-concentrate diet supplemented with capsaicin.

In a PCoA analysis, clear separation was evident among these three groups (Figure 2A), with PC1 and PC2, respectively, explaining 49.48% and 25.41% of the total change. Greater separation was noted between the CON and SARA groups. In total, 1145 OTUs were common across all three groups, while 421, 42, and 109 were uniquely present in samples from the CON, SARA, and CAP groups, respectively (Figure 2B).

**Figure 3:**
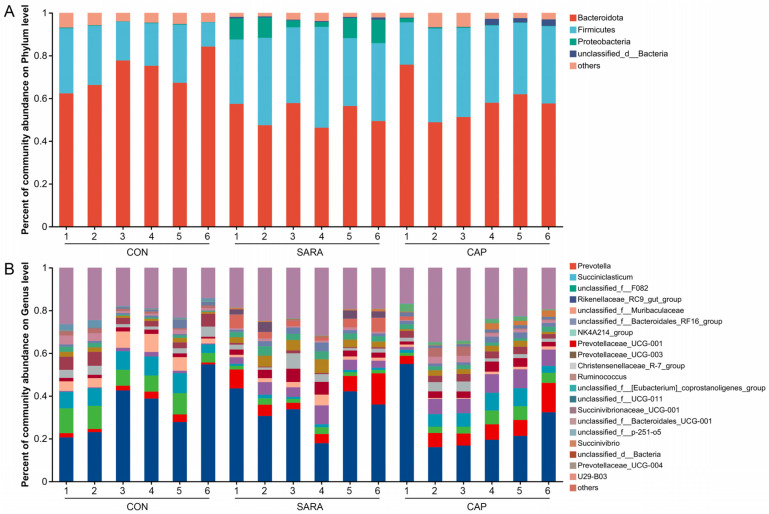
The relative abundance of ruminal bacteria in the indicated treatment groups at the (**A**) phylum and (**B**) genus levels. CON, control diet; SARA, high-concentrate diet; CAP, high-concentrate diet supplemented with capsaicin.

The relative bacterial abundance at the phylum and genus levels is presented in Figure 3. The dominant phyla across all groups were Bacteroidota and Firmicutes. In the CON group, the relative abundance levels of Bacteroidota and Firmicutes were 72.16% and 22.43%, respectively, as compared to 52.44% and 36.94% in the SARA group, and 58.85% and 35.18% in the CON group (Supplementary Table S2). *Prevotella* was the most abundant bacterial genus, accounting for 34.58%, 33.98%, and 26.81% of the genera in the CON, SARA, and CAP groups, respectively (Supplementary Table S3). The second most abundant genera in the CON group was *unclassified_f__F082* (8.63%), whereas *Succiniclasticum* was the second most abundant in the SARA (7.21%) and CAP (7.43%) groups.

**Figure 4:**
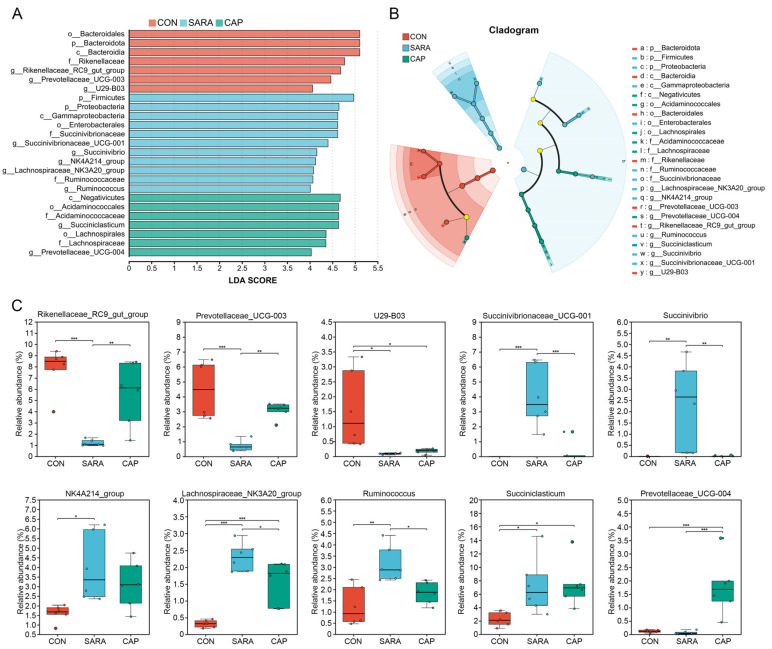
Differences in the composition of the ruminal microbiome in the three treatment groups. (**A**) Linear discriminant analysis (LDA) scores for significantly changed bacteria in different treatment groups as assessed via the linear discriminant analysis effect size (LEfSe) method. A threshold logarithmic LDA score for discriminative features of 4.0 was used for these analyses. (**B**) A cladogram of bacteria exhibiting significant differential abundance. Differences are represented in the color of the most abundant taxa. (**C**) A box plot showing ruminal bacteria that were significantly differentially abundant. CON, control diet; SARA, high-concentrate diet; CAP, high-concentrate diet supplemented with capsaicin. * *p* < 0.05; ** *p* < 0.01, *** *p* < 0.001.

The ruminal bacteria was further analyzed at the phylum and genus levels through a LEfSe approach, screening out unique bacteria exhibiting LDA scores > 4 (Figure 4A). The taxonomic cladogram derived from this LEfSe analysis is presented in Figure 4B. The unique genera of bacteria identified in the CON group included *Rikenellaceae_RC9_gut_group*, *Prevotellaceae_UCG-003*, and *U29-B03*, with significantly greater abundance as compared to the other groups (*p* < 0.05) (Figure 4C). The unique bacteria identified in the SARA group included *Succinivibrionaceae_UCG-001*, *Succinivibrio*, *NK4A214_group*, *Lachnospiraceae_NK3A20_group*, and *Ruminococcus*, while unique genera for samples from the CAP group included *Succiniclasticum* and *Prevotellaceae_UCG-004*.

### 3.4. Correlation Analyses

**Figure 5:**
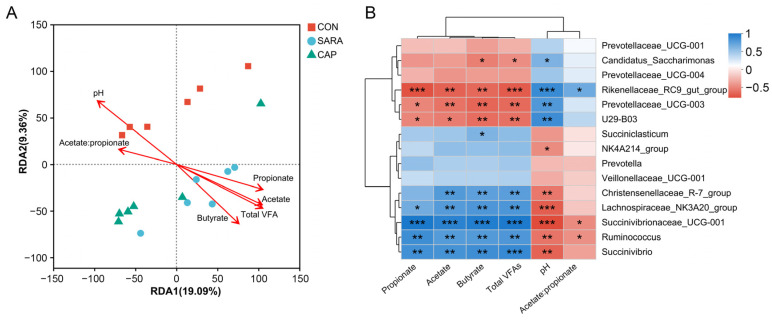
Correlation analysis between community structure and environmental factors. (**A**) Redundancy analysis (RDA) of bacterial data (symbols) and ruminal fermentation parameters (arrows). (**B**) A correlation matrix showing the relationships between ruminal fermentation parameters and the genus-level composition of the ruminal bacteria. Positive and negative correlations are, respectively, represented in blue and red, with color intensity being proportional to the Pearson correlation coefficient. CON, control diet; SARA, high-concentrate diet; CAP, high-concentrate diet supplemented with capsaicin. * *p* < 0.05; ** *p* < 0.01, *** *p* < 0.001.

RDA analyses indicated that the structure of the ruminal bacterial community is significantly influenced by ruminal pH, total VFA, acetic acid, propionic acid, butyric acid, and the acetate-to-propionate ratio levels (*p* < 0.05, Figure 5A). In the CON group, samples were positively correlated with ruminal pH and the acetate-to-propionate ratio, whereas the opposite was true for samples from the SARA and CAP groups.

Spearman’s rank correlation analyses revealed positive correlations between *Christensenellaceae_R-7_group*, *Lachnospiraceae_NK3A20_group*, *Succinivibrionaceae_UCG-001*, *Ruminococcus*, and *Succinivibrio* levels and total VFAs, acetate, propionate, and butyrate concentrations (*p* < 0.05, Figure 5B), whereas they were negatively correlated with ruminal pH (*p* < 0.05). In contrast, the opposite trend was evident for the *Rikenellaceae_RC9_gut_group*, *Prevotellaceae_UCG-003*, and *U29-B03*.

## 4. Discussion

Ruminal health is an extremely important consideration for healthy and efficient beef cattle production. To meet the nutritional requirements necessary to achieve rapid and cost-effective growth, high-concentrate diets are commonly used to feed beef cattle. A sustained high-grain diet intake in dairy cows, however, triggers abnormal fermentation in the rumen that is characterized by the production of excessively high levels of VFAs [33]. When the production of these organic acids exceeds the capacity of the rumen to absorb and/or buffer them, they will accumulate, leading to a reduction in ruminal pH and concomitant microbial dysbiosis that can affect the health of ruminants [34,35,36]. In this study, the effects of a high-concentrate diet on fermentation-related parameters and the composition of the microbiome in the rumen were evaluated in beef cattle, with CAP further being tested as a potential dietary supplement capable of mitigating the negative impacts of high-concentrate diet consumption. The overall goal of this approach was to establish novel approaches to preventing SARA onset and associated losses in the context of beef cattle production.

For these analyses, a beef cattle model of SARA was established using a high-concentrate (80% concentrate) diet. SARA is often diagnosed based on a low ruminal pH [37]. During the early stages of onset, however, SARA tends to exhibit inconsistent and atypical symptoms, such that the most appropriate diagnostic threshold value for ruminal pH remains uncertain [38,39]. Plaizier [40], for instance, proposed a pH threshold of 6.0 for SARA diagnosis when analyzing ruminal fluid samples collected via stomach tube, whereas Garrett et al. [41] collected samples via rumenocentesis, and proposed a threshold pH of 5.5. Using a pH threshold of 5.5 and continuous ruminal pH measurements at the base of the cranial ventral sac, Schwaiger et al. [42] similarly proposed that cattle face an elevated risk of SARA. The timing of ruminal fluid sampling relative to feeding can also influence the accuracy of pH measurements, given that these pH levels can fluctuate over the course of the day. In some studies, a higher risk of SARA has been reported when the ruminal pH falls below 5.6 for more than 3 h per day [43], or below 5.8 for more than 5–6 h per day [44]. These differing ruminal pH thresholds used to diagnose SARA in various studies may be related to the sensitivity of pH measurements to the timing of measurements, the chosen measurement approach, and the sampled site within the rumen [45]. It is, thus, crucial that more accurate approaches to precisely diagnosing and preventatively managing SARA risk be established. Here, a diet consisting of 80% concentrate was used to induce SARA, with this concentration being higher than that reported previously [46,47,48]. Consistently, the pH of the rumen among cattle in the SARA group was as low as 5.4, supporting the successful establishment of the SARA model system.

SARA model cattle exhibited the expected reductions in ruminal pH and acetate/propionate ratio, together with increases in total VFA, acetate, and propionate concentrations relative to the CON group, in line with past reports [49,50]. The inclusion of higher levels of fermentable carbohydrates in grain-rich diets generally takes place at the expense of fiber content, particularly physically effective neutral detergent fiber (peNDF). These carbohydrates can be quickly fermented by microbes in the rumen, generating large volumes of VFAs, and thereby reducing the local pH [37]. The reduced peNDF content also limits the chewing activity of these beef cattle, decreasing the amount of secreted saliva necessary to neutralize VFAs and reducing ruminal peristalsis, as well as the rate of ruminal passage [51]. High-grain diet-induced SARA thus influences ruminal fermentation activity while also having broader implications for the overall health of the rumen. Notably, CAP was able to abrogate the adverse effects of SARA modeling on the measured ruminal fermentation parameters through increases in pH levels and reduced VFA levels in the rumen. These effects may be attributable to the ability of CAP to enhance the secretion of saliva, thus leading to VFA neutralization, and/or to its ability to promote rumen motility, leading to improved VFA absorption by the rumen epithelium and accelerating the passage rate for ruminal contents. CAP serves as a specific TRPV1 agonist, a discovery for which the 2021 Nobel Prize in Physiology or Medicine was awarded [52]. In rabbits and mice, CAP has been shown to specifically activate TRPV1, leading to increased paracellular pathway permeability in the salivary epithelium, and resulting in higher levels of secretory output [19,20,21]. CAP can also reportedly improve swallowing functions in humans [53], while in rats, it can stimulate intestinal activity [54]. CAP can, therefore, positively affect fermentation in the rumen. However, additional research remains necessary to fully clarify the mechanistic basis for these effects.

As the largest and most important digestive organ found in ruminants, the rumen functions similarly to an anaerobic biological fermentation tank, and is home to a diverse community of bacteria, fungi, protozoa, archaea, and viruses. Of these microorganisms, ruminal bacteria are considered the most abundant and most important, with upwards of 10^11^ bacterial cells per mL of ruminal fluid [55]. These ruminal bacteria are essential regulators of ruminant physiology, shaping digestion, absorption, neuroendocrine activity, metabolism, and immunological homeostasis [26]. Next-generation sequencing technologies have fueled growing research interest in the 16S rRNA sequencing of the microflora within the rumen [56,57]. Here, ruminal samples from the cattle in the SARA group exhibited reductions in bacterial richness and diversity relative to the CON group, consistent with past reports [58,59]. This decline in alpha diversity also coincided with the presence of fewer unique bacterial OTUs in the SARA group, relative to CON samples. These effects may be a consequence of SARA-related acidification, and the consequent death of many ruminal microbes that are not equipped to tolerate low pH levels [60]. Relative to the SARA group, the ruminal bacteria of CAP-treated cattle exhibited increased richness and diversity, together with a higher total number of unique OTUs. PCoA analyses also revealed a clear separation between samples from the CAP and SARA groups, confirming the ability of CAP supplementation to reshape the structure of the bacterial community present within the rumen.

The bacterial community within the rumen is dynamic and complex, and can respond collectively to various factors, including health, environment, dietary composition, and cattle breed [61]. Diet, in particular, may play a central role in shaping the structure and function of bacterial communities [62]. Bacteroidetes and Firmicutes were found to be the most abundant bacterial phyla in the rumen, followed by Proteobacteria, with the former two comprising ~90% of all ruminal bacteria. Bacteroidetes and Firmicutes, thus, appear to be the main core microbial communities within the rumen, consistent with past reports [46,47]. Bacteroidetes abundance was reduced in the SARA group relative to the CON group, whereas Firmicutes and Proteobacteria abundance increased in these animals. Similar trends have also been observed in dairy cows [47] and sheep [63] with SARA. At the genus level, *Prevotella* accounted for more than 25% of the bacteria in each group by abundance, while the relative abundance levels for other genera were <9%, suggesting that *Prevotella* is the most abundant bacterial genera in beef cattle. *Prevotella* is reportedly widely present throughout the rumen and gastrointestinal tracts of herbivores and omnivores, wherein it can break down fiber, starch, and protein [64]. LEfSe analyses were also used to explore unique ruminal bacteria that may offer value as candidate biomarkers suitable for differentiating among the groups in this study (LDA scores > 4). This approach revealed the enrichment of *Succinivibrionaceae_UCG-001*, *Succinivibrio*, *NK4A214_group*, *Lachnospiraceae_NK3A20_group*, and *Ruminococcus* in the SARA group, such that they may offer value as markers for SARA diagnosis. Additional research, however, will be required to validate this hypothesis. The enrichment of *Succiniclasticum* and *Prevotellaceae_UCG-004*, in contrast, was observed in the CAP group. *Succiniclasticum*, a member of the Firmicutes phylum, serves as the predominant succinic acid-producing bacterium in the rumen and plays a vital role in the conversion of succinate to propionate, which is the major precursor to gluconeogenesis in ruminants [65]. Wang et al. [66] reported that *Succiniclasticum* can help stabilize the rumen environment through the conversion of succinate into propionate, particularly in ruminants fed a starch-rich high-energy diet. However, it was reported that a decrease in propionic acid production was accompanied by a decrease in the relative abundance of *Succiniclasticum* [67], which is contrary to the results of this study. The reason for these different results might be that CAP promoted salivary secretion and then neutralized the acid in the rumen. Unfortunately, the function of Succiniclasticum in the rumen remains enigmatic. The specific role of *Succiniclasticum* on rumen metabolism needs to be further explored. *Prevotellaceae_UCG-004* is a member of the Prevotellaceae family, which has been reported to be positively correlated with ruminal fermentation patterns and feed efficiency [68]. It is also important to note that the relative abundance of individual bacterial phyla and genera in SARA model cattle tended to match those in the CON group more closely following CAP supplementation, in line with the observed shifts in ruminal pH. These results suggest that CAP can abrogate the pathogenesis of SARA through the maintenance of a more stable ruminal bacterial community.

In an RDA, ruminal fermentation parameters were found to have a clear effect on the bacterial community within the rumen. Longer arrows were evident for ruminal pH and total VFAs across all groups, suggesting that these factors most strongly affected bacterial communities, as expected. Spearman’s rank correlation analyses further revealed the enrichment of *Rikenellaceae_RC9_gut_group*, *Prevotellaceae_UCG-003*, and *U29-B03* in the CON group, with these microbes being negatively correlated with total and individual VFA levels, whereas they were positively correlated with ruminal pH. *Rikenellaceae_RC9_gut_group* and *Prevotellaceae_UCG-003* have also previously been reported to be negatively correlated with ruminal fermentation parameters in vitro, including total VFA, butyrate, acetate, and propionate concentrations [69]. *Rikenellaceae RC9 gut group* also plays central roles in the digestion of fiber, the ruminal fermentation pattern, and rumen epithelial development [68]. *U29-B03* is a member of the relatively newly identified Rikenellaceae family, which generally ferments proteins or carbohydrates, although its precise metabolic functions require further study [47,70]. The SARA group, in contrast, exhibited *Lachnospiraceae_NK3A20_group*, *Succinivibrionaceae_UCG-001*, *Ruminococcus*, and *Succinivibrio* enrichment, with these changes being positively correlated with the levels of total VFAs and individual VFAs in the rumen, while they were negatively correlated with ruminal pH. *Lachnospiraceae_NK3A20_group*, the most prevalent Firmicutes genus, can hydrolyze starch and decompose cellulose, resulting in the production of compounds including formate and acetate [71]. *Succinivibrionaceae UCG-001* and *Succinivibrio* are major members of the Succinivibrionaceae family that can limit methane emissions through the production of succinate, thereby trapping rather than releasing metabolic hydrogen [72]. *Ruminococcus* species are vital mediators of the digestion of resistant starch, and they have also been tentatively linked to metabolic pathways associated with a range of immunological, intestinal, and neurological disorders [73]. Together, these results suggest that diet-induced variability in ruminal fermentation patterns is mediated primarily by changes in the makeup of the bacterial community found within the rumen.

## 5. Conclusions

This study demonstrated that the SARA was successfully induced by the high-grain diet, which subsequently reduced the richness and diversity of ruminal bacteria. The supplementation of CAP alleviated the negative impact on rumen internal environment caused by SARA, via elevating the ruminal pH and decreasing the concentration of total VFAs. Interestingly, the supplementation of CAP in these SARA model cattle tended to alter the composition of the ruminal bacteria to more closely mirror that of control cattle. Furthermore, the addition of CAP resulted in an increased relative abundance of *Succiniclasticum* and *Prevotellaceae_UCG-004*, both of which are positively associated with ruminal homeostasis and feed efficiency. These results indicated that CAP not only attenuated adverse SARA-related effects but also played an important role in the restoration of ruminal microbial community.

## Figures and Tables

**Table 1:** Experimental diet ingredients and chemical composition.

Items	CON	SARA
Ingredients, % of DM		
Corn grain	8.35	57.65
Soybean meal	6.35	10.95
Wheat bran	2.10	8.21
Corn straw silage	53.28	13.31
Wheat straw	26.72	6.68
CaHPO_4_	0.80	0.80
CaCO_3_	0.80	0.80
NaCl	0.80	0.80
Premix ^1^	0.80	0.80
Chemical composition ^2^		
CP, % of DM	8.87	12.81
NDF, % of DM	59.07	24.88
ADF, % of DM	41.69	14.38
Ash, % of DM	8.83	4.06
ME, MJ/kg	7.96	11.38
NEm, MJ/kg	4.47	7.51
NEg, MJ/kg	2.19	4.91

^1^ Each kg of premix contains VA 2000 KIU, VD_3_ 800 KIU, VE 2000 mg, Cu 3 g, Fe 30 g, Mn 25 g, Zn 24 g, I 500 mg, Se 100 mg, Co 50 mg. ^2^ DM, dry matter; CP, crude protein; NDF, neutral detergent fiber; ADF, acid detergent fiber. ME, NEm and NEg were calculated values, while other values were measured.

**Table 2:** Effects of treatment on ruminal pH and VFAs in beef cattle.

Item	CON	SARA	CAP	SEM	*p*-Value
pH	6.61 ^a^	5.40 ^b^	6.36 ^a^	0.145	<0.001
VFAs, mmol/L					
Total VFA	74.48 ^b^	133.95 ^a^	82.86 ^b^	7.728	<0.001
Acetate	49.71 ^b^	77.06 ^a^	52.11 ^b^	3.810	<0.001
Propionate	16.09 ^b^	36.76 ^a^	17.90 ^b^	2.998	0.002
Butyrate	5.74 ^b^	15.32 ^a^	7.67 ^b^	1.345	0.002
Isobutyrate	1.15	0.94	1.05	0.161	0.887
Valerate	0.57 ^b^	1.59 ^a^	1.25 ^a^	0.136	0.002
Isovalerate	1.15 ^b^	1.93 ^ab^	2.73 ^a^	0.222	0.006
Acetate/propionate	3.28	2.34	3.03	0.199	0.132

Different lowercase letters in the same row indicate significant differences in mean values (*p* < 0.05). CON, control diet; SARA, high-concentrate diet; CAP, high-concentrate diet supplemented with capsaicin.

## Data Availability

The 16S rRNA sequence data were submitted to the NCBI Sequence Read Archive (SRA; https://submit.ncbi.nlm.nih.gov/subs/sra/; accessed on 26 December 2024) database with the accession number of PRJNA1182293 for open access.

## Supplementary Materials

The following supporting information can be downloaded at: https://www.mdpi.com/article/10.3390/microorganisms13010084/s1, Table S1: Alpha diversity indices of the bacterial community in all samples; Table S2: Relative abundance of bacterial community in all samples at phylum level; Table S3: Relative abundance of bacterial community in all samples at genus level.
